# Quantification of system resilience through stress testing using a predictive analysis of departure dynamics in a $M^X/G/1/N$ queue with multiple vacation policy

**DOI:** 10.1038/s41598-025-95478-w

**Published:** 2025-03-26

**Authors:** Rafał Marjasz, Wojciech M. Kempa, Viacheslav Kovtun

**Affiliations:** 1 Institute of Theoretical and Applied Informatics, Polish Academy of Sciences, Bałtycka 5, 44-100 Gliwice, Poland; 2 Faculty of Applied Mathematics, Silesian University of Technology, 23 Kaszubska Str., 44-100 Gliwice, Poland; 3 Faculty of Intelligent Information Technologies and Automation, Department of Computer Control Systems, Vinnytsia National Technical University, 95 Khmelnitske Shose Str., Vinnytsia, 21000 Ukraine

**Keywords:** Departure process, Finite buffer, Integral equations, Multiple vacation, Transient state, Stress testing, System resilience, Applied mathematics, Computer science, Information technology

## Abstract

The study investigates the departure counting process in a finite-buffer queueing system with batch arrivals and multiple vacation policy, focusing on quantifying system resilience through stress testing and predictive analysis. A representation for the mixed double transform of the number of departures up to a fixed time moment is obtained in explicit form by applying an analytic approach based on integral equations and linear algebra. We perform a comparative analysis of numerical calculations and simulations made in OMNeT++ Discrete Event Simulator. The attached numerical study aims to understand how the queueing system copes under challenging conditions, examining the impact of various system parameters on the behaviour of the mean number of packets processed within a fixed time frame. Utilizing numerical experiments, the study analyzes the influence of vacation duration, initial buffer state, arrival intensity, and processing rate on the departure process. This enables the understanding of system recovery dynamics, particularly in how critical infrastructures can be optimized for resilience against disruptions. Results reveal significant dependencies between these parameters and the transient behaviour of the queueing system. Notably, the service speed parameter demonstrates the most substantial influence on the mean number of processed packets, followed by the arrival rate. Conversely, variations in vacation duration and initial packet count exhibit comparatively minor effects on system behaviour. Overall, the findings provide valuable insights into the dynamics of departure processes in finite-buffer queue systems with batch arrivals and multiple vacation policies, offering implications for system optimization, performance enhancement strategies, and resilience assessment in the face of potential system failures or disasters.

## Introduction and motivation

Currently, in modeling technological solutions conducive to energy saving, queueing models with various types of mechanisms limiting the availability of service stations are often used. Analytical results obtained for such models are used in the design of network protocols in wireless transmission, algorithms that improve the efficiency of production processes and solutions that redo the throughput of important transportation nodes and logistics centers.

Among many solutions found in the literature, the discipline of multiple vacation can be distinguished. In this policy, the service station is deactivated when the system is emptied of packages requiring processing (i.e. when the busy period ends). At this point, the server vacation period begins (of random or fixed length). After its completion, the state of the buffer accumulating incoming packets is monitored. If it is non-empty (at least one packet needs to be processed), the server immediately returns to serving packets. Otherwise (if the buffer is empty) next server vacation period begins and so on. In general, within the multiple vacation policy, the number of consecutive independent server vacation periods is unlimited.

In practice, multiple vacation policy can be considered not only as a solution enabling, for example, repairs or periodic technical inspections of service stations. During the period of suspension of service (vacation period), the service station may perform other (secondary) tasks, which it abandons when“basic” tasks appear. This mechanism can therefore support the effective use of the service station through periodic monitoring of the state of the accumulating buffer.

One of the key stochastic characteristics of the queueing model is the departure process, i.e. the process counting handled packets until a specified moment in time. Obviously, this characteristic is an important determinant of the system operation, determining its throughput. In the paper, we consider a queueing model with a multiple vacation mechanism limiting access to the service station. Investigating this characteristic can have the following practical applications which are, simultaneously, motivations for the analysis:

- Computer and telecommunication networks: the throughput (expressed via the departure process) of the selected network node (on the route the packet takes from host A to host B) is crucial in assessing the quality of the network (QoS), i.e. packet delivery time, risk of packet loss due to buffer overflow, etc.;
- Production lines: knowledge of the behavior of the departure process of a given element (station, machine, etc.) allows for more effective management of the production process, including planning the appropriate size of accumulating buffers at the next stations;
- Road junctions (transportation): the “throughput” of a given crossroad in the city communication system has an obvious significant impact on the capacity of the entire communication system (traffic jams at a given crossroad are most often then “inherited” by subsequent crossroads).

Most of the analytical results for various queueing models are related to the steady-state (stationary) stochastic characteristics of these models.

In practice, however, the steady state does not always reflect the system operation well. This is the case, for example, when traffic control mechanisms or system input parameters characterizing the rate of packet arrival or the speed of their processing are frequently changed. Obviously, the analysis of the functioning of the system in the initial period of its operation requires the use of a transient rather than a stationary state. This paper aims to address this gap by examining the departure process in a non-stationary state of the system, particularly under challenging conditions or even potential disasters. By focusing on quantifying system resilience through stress testing and predictive analysis, we investigate how the queueing system copes with non-standard situations related to overloads caused by potential failures or computer network disruptions. Our approach provides valuable insights into the dynamics of departure processes, offering implications for system optimization and performance enhancement studies, especially in the face of adversities.

In the article, we consider a stochastic process that counts handled packets (departure process, output process) in the non-stationary state of the system, i.e. at a fixed time *t*. In the considered model, we assume that packets appear in groups of random size in accordance with the Poisson process with constant intensity. Packets are handled individually according to a general-type probability distribution. The buffer accumulating incoming packets has a limited capacity, which has practical reasons. In real service systems, the waiting room usually has a finite capacity (e.g., in network switches, buffers of production systems, in parking lots, etc.). This means that packets that arrive in the system during the period in which the service station is occupied and the accumulation buffer is completely full are lost.

The remaining part of the paper is organized as follows. In State of the Art section, we present the review of bibliographic items concerning queueing models with server vacations and results on departure process. The Queueing system section presents a precise mathematical description of the considered queueing model. In the next section, we build a system of integral equations for conditional transient departure process and the corresponding system written for Laplace transforms. In section Departure process solution in a compact form, we obtain a solution of the system written for Laplace transforms in a compact form. In the following section, a representation for the conditional mean number of departures up to the fixed time moment is derived. Section Numerical examples contains numerical examples illustrating the impact of key system parameters on the behavior of the mean number of departures until the fixed time epoch. The last section contains a brief conclusion.

## State of the art

As it seems, probably the first formalized analytic result concerning the departure process in a queueing system can be found in^1^. It it shown there that in an *M*/*M*/*c*-type queue the departure process of packets forms a Poisson stream. In^2^ mathematical closed-formulae for the output process in such a model are obtained. The stationary-state departure process in an *M*/*G*/1 system is considered in^3^ where the formula for the Laplace-Stieltjes transform of inter-departure time probability distribution is obtained. A first general study on results derived for departure process in queueing systems is presented in^4^. This survey is then continued in^5^ where new results for asymptotic behavior of the output process are discussed.

A Wiener-Hopf factorization technique is applied in^6^ to find the formula for the double transform of departure process in a general-type *GI*/*G*/1 queue with group arrivals. A discrete-type queueing model is investigated in^7^ where a unified matrix-analytic method is used for the analysis of departure process. One can find an interesting example of applying a queueing model in the study of the airport departure process in^8^. In^9^ the departure process in a queueing model with a *MAP*-type auto-correlated input flow of packets and Markovian service process is considered. It is proved there that even when an arrival process is short-range dependent, the departure process could has long-range dependence if a service-time process is long-range dependent. A more general-type model with *BMAP*-type batch-arrival process is investigated in^10^. In^11^ the output process of packets in a finite-buffer birth-and-death Markovian queueing model is analyzed. It is proved there that if the birth rates are non-increasing and the death rates are non-decreasing the limiting index of dispersion of counts of the output process is less than one. In^12^ the departure process in a queueing model with unbounded number of servers and a Markov arrival process is studied. It is shown there that for growing service duration, the output stream is asymptotically a simple stream independent of the service duration distribution.

One can find in^13^ am extended study on analytic results concerning queueing models with various types of vacation policies. In^14^ an *M*/*G*/1-type queueing system with batch arrival of packets and single vacation policy is considered. Representations for the mean number of departures occurring in a fixed-length time interval and its asymptotic expansion are obtained by using a probability decomposition method. A factorization-based approach is used for the analysis of departure process in^15^ in the case of the $M^{X}/G/1$-type model with single server vacations of exhaustive type. In^16^ a discrete-time finite-capacity queueing model operating under multiple vacation policy is studied. The arrival process is governed by a general discrete-time renewal process while service and vacation times are geometrically distributed. Representations for the stationary queue-size distribution at different time epochs are found there. A batch-arrival queueing system with Poisson input stream and general service times with disasters and repairs under a multiple adapted vacation policy is analyzed in^17^. A queueing model with multiple machine vacations and with state-dependent arrival process is investigated in^18^. Steady-state results are obtained via matrix geometric approach. In^19^ a queueing system with batch customer arrivals, multiple server vacations, N-policy, balking and control policy on request for re-service is considered. An *M*/*M*/1-type system with multiple differentiated vacations, impatient customers and a waiting service station is investigated in^20^. By using probability generating function technique the formulae for transient system state probabilities are found there. In^21^ an *M*/*G*/1-type clearing queueing model with server setups and multiple vacations is investigated, in which all present customers in the system are being processed simultaneously and breakdowns may occur during busy or setup period. The formulae for the queue-size and sojourn time distributions in the equilibrium are found. A bulk arrival and batch service queueing system with server failures and multiple vacation policy is considered in^22^. The representation for the probability generating function of the queue-size distribution is obtained via supplementary variable technique. In^23^ an infinite-buffer discrete-time queueing model with server setup/close-down times and multiple vacation policy is analyzed. A model with multiple vacations and a threshold-type policy is proposed in^24^ to study power-saving mechanisms in wireless sensor networks using the dynamic power management technique. The model considers four system states: a busy state, wake-up state, shutdown state and inactive state. In^25^ a finite-buffer with single and multiple server vacations is investigated in which the arrival process is a Markovian Arrival Process (MAP) and service and vacation times are generally distributed. The formulae for the stationary queue-size distribution at different-type epochs are obtained there. An $M^{X}/G/1$-type queueing system with multiple server vacations is considered in^26^ in the transient state. A compact-form representation for the double transform of departure process is obtained applying the approach based on integral equations, Wiener-Hopf factorization and renewal theory (see also^27^)). In^28^ the explicit formulae for the transient queueing delay distribution in a finite-buffer *M*/*G*/1-type model with single and multiple vacations are found. A model with general independent input flow and server working vacation policy is considered in^29^.

In^30^, the problem of inter-departure time correlations in PH/G/1-type queueing models is investigated in the context of non-Markovian tandem queueing networks, in which the output process of one service station becomes the arrival process to another one. An interesting problem of modeling the arrival and departure stochastic processes at the transport node is studied in^31^ in the form of a case study of an international airport. In^32^, the law of the iterated logarithm for the performance measures of a two-station queueing network with independent arrivals is developed by a strong approximation method. For the queueing network considered in heavy traffic, in particular, the law of the iterated logarithm for the departure process is obtained and represented by some simple functions. One can find in^33^ a machine learning-based approach for predicting the taxi-out time, with the departure process decomposed into two components: the time taken to travel from the gate to the departure queue and the time spent in the departure queue. In^34^, the problem of re-direction in a queueing network consisting of single-server stations serving two customer types with different service time requirements is analyzed. Re-direction occurs when a customer arriving at a station in a queuing network has to be re-directed to a downstream station to complete the service. The stochastic departure process (and, as a consequence, the arrival process to downstream stations) is non-Markovian, even when all external arrivals and all services are Markovian.

## Queueing system

We consider an $M^X/G/1/N$-type queueing system in which packets arrive in batches (groups) according to a compound Poisson process with rate $\lambda$ batches per time unit. The size of an arriving group is a random variable and $p_{k}$ denotes the probability that a group consists of *k* packets, where $\sum _{k=1}^{\infty }p_{k}=1$. Packets are processed individually according to the FIFO service discipline with generally distributed service times specified by the cumulative distribution function (CDF) $F(\cdot )$ and Laplace-Stieltjes transform (LST) $f(\cdot )$. The value *N* determines the maximal number of packets allowed to be present in the system simultaneously, which means that there are $N-1$ places in the buffer queue and one place in the processing station. A multiple vacation period (MVP) is initialized whenever the system becomes empty. During this period, the successive vacations are being started, one by one, until at least one packet arrives in the buffer and is detected at the completion epoch of one of those vacations. All the vacations are assumed to be independent and identically distributed random variables with a common general-type CDF $G(\cdot )$ with LST $g(\cdot )$.

Denote by *h*(*t*) a (random) number of packets completely processed up to the fixed time *t*. Let us define the conditional distribution of the number of packets handled until time $t>0$, on the condition that the number of packets accumulated in the buffer at the initial time $t=0$ equals *n* ($Y(0)=n$) as follows:

1 $$\begin{aligned} &H_{n}^X(t,m)={\textbf{P}}\{h(t)=m\,|\,Y(0)=n\},\quad t>0,\,0\le n\le N, \end{aligned}$$

where, according to Kendall’s notation, the superscript *X* refers to the batch inflow of packets.

We will be interested in the explicit compact-form representation for the double transform of the probability distribution of *h*(*t*), i.e. for the functional

2 $$\begin{aligned} &\widetilde{h}_{n}^X(s,z){\mathop {=}\limits ^{def}}\sum _{m=0}^{\infty }z^{m}\int _{0}^{\infty }e^{-st}H_{n}^X(t,m)dt \end{aligned}$$

where $$0\le n\le N,\,|z|<1,\,\textrm{Re}(s)>0$$.

The following theorem can be found in^35^ (see also^36^). Its essence is the ability to write in a compact form the solution of a specific system of linear equations.

### Theorem 1

Let there be two functional sequences $(a_n(s))$ and $(\psi _n(s,y))$, with $a_0(s)\ne 0.$ Each solution of the system of equations of the form

3 $$\begin{aligned} &\sum _{k=-1}^{n}a_{k+1}(s)x_{n-k}(s,y)-x_n(s,y)=\psi _n(s,y),\hspace{0.7cm} n\ge 0 \end{aligned}$$

can be expressed in the following form:

4 $$\begin{aligned} &x_{n}(s,y)=C(s,y)R_{n+1}(s)+\sum _{k=0}^{n}R_{n-k}(s)\psi _k(s,y), \hspace{0.7cm} n\ge 0 \end{aligned}$$

where *C*(*s*, *y*) does not depend on *n*,  and the sequence $R_k(s)$ is defined as follows

5 $$\begin{aligned} &R_0(s)=0, \hspace{0.1cm} R_1(s)=a_0^{-1}(s), \hspace{0.1cm} R_{k+1}(s)=R_1(s)\left[ R_k(s)-\sum _{i=0}^{k}a_{i+1}(s)R_{k-i}(s)\right] \end{aligned}$$

for $$k\ge 1.$$

In the following sections, applying the law of total probability, the embedded Markov chain paradigm, using Volterra-type integral equations and the formula 4, we will obtain representation for the mixed double transform, namely for the probability generating function (PGF) of the Laplace transform (LT) of *h*(*t*), conditioned by the initial buffer state.

## Conditional departure process integral equations

In the present section, using the idea of an embedded Markov chain, we build a system of integral equations for the conditional departure process. Then, we convert it to an appropriate system written for double-mixed transforms and rewrite it in a specific form.

Let us first assume that the system is empty at startup, so MVP starts at $t=0.$ Utilizing the notation introduced in (1), the formula of total probability leads to the following Volterra-type integral equation:

6 $$\begin{aligned} &H_{0}^X(t,m)=\sum _{k=1}^{N-1}p_k\sum _{i=0}^{\infty }\int _{u=0}^{t}dG^{i*}(u)\int _{y=u}^{t}\lambda e^{-\lambda y}dy\int _{v=y-u}^{t-u} \Biggl [\sum _{r=0}^{N-k-1}\sum _{j=0}^{r}p_r^{j*}\nonumber \\&\frac{\bigl [\lambda (u+v-y)\bigr ]^{j}}{j!}e^{-\lambda (u+v-y)}H_{k+r}^X(t-u-v,m)\nonumber \\ &+H_{N}^X(t-u-v,m)\sum _{r=N-k}^{\infty }\sum _{j=0}^{r}p_r^{j*}\frac{\bigl [\lambda (u+v-y)\bigr ]^{j}}{j!}e^{-\lambda (u+v-y)}\Biggr ]dG(v)+\sum _{k=N}^{\infty }p_k\sum _{i=0}^{\infty }\int _{u=0}^{t}dG^{i*}(u)\int _{y=u}^{t}\lambda e^{-\lambda y}dy \nonumber \\ &\times \int _{v=y-u}^{t-u}H_{N}^X(t-u-v,m)dG(v)+\sum _{i=0}^{\infty }\int _{u=0}^{t}\delta _{m,0}\overline{G}(t-u)dG^{i*}(u)\int _{y=u}^{t}\lambda e^{-\lambda y}dy+\delta _{m,0}e^{-\lambda t}, \end{aligned}$$

where $G^{i*}(\cdot )$ stands for the *i*-fold Stieltjes convolution of the cumulative distribution function $G(\cdot )$ with itself, $p_{i}^{j*}$ denotes the *i*th term of the *j*-fold convolution of the sequence $(p_{k})$ with itself, $\delta _{i,j}$ denotes the Kronecker delta function and $\overline{G}(t){\mathop {=}\limits ^{def}}1-G(t)$.

Let us comment (6) briefly. The first element of the sum corresponds to the situation in which the MVP ends at time *t*. At the moment of its end, the number of packets accumulated in the buffer does not exceed its capacity. In such a case, the handling of packets starts after the end of the last vacation period of length *v* (at the moment $u+v<t$ corresponding to the total length of the initial MVP), and $k+r$ packets may be accumulated in the buffer, where *k* is the number of packets arrived at time *y*, and $r\in \{0,\cdots , N-k-1\}$ is the number of packets that arrived in the system in a time interval of length $u+v-y$. The second and third components of the sum describe the case in which the buffer is saturated during the MVP ending precisely at time *t*. The $H_{N}^X(t-u-v,m)$ function present in both components the situation in which at the moment $u+v<t$ the server starts processing with exactly *N* packets accumulated in the system. The difference in these components is in how the buffer becomes saturated. The second component implements the case of multiple (at least twice) group inflow of packets, the number of which, when added up, gives a value equal to at least *N*. The third component refers to the case in which a single inflow of a group of packets saturates the buffer.

The fourth component on the right side of (6) reflects the case of the arrival of the first packet before time *t*,  resulting in the start of the service process (i.e., the end of the MVP) following the moment *t* (at the moment $u+v>t$). In contrast, the fifth component corresponds to the first packet arriving in the system after time *t*.

Secondly, we consider the case of the nonempty system at the starting time $t=0.$ We take advantage of the fact that in the evolution of the $M^X/\cdot /\cdot$ FIFO queue, the successive departure epochs are Markov moments. Therefore, applying the total probability law with respect to the first departure epoch after $t=0,$ we get:

7 $$\begin{aligned} &H_{n}^X(t,m)=I\{m\ge 1\}\int _{0}^{t}\Bigl [\sum _{k=0}^{N-n-1}\sum _{j=0}^{k}p_k^{j*}\frac{(\lambda y)^{j}}{j!}e^{-\lambda y}H_{n+k-1}^X(t-y,m-1)\nonumber \\ &+H_{N-1}^X(t-y,m-1)\sum _{k=N-n}^{\infty }\sum _{j=0}^{k}p_k^{j*}\frac{(\lambda y)^{j}}{j!}e^{-\lambda y}\Bigr ]dF(y)+\delta _{m,0}\overline{F}(t), \end{aligned}$$

where $\overline{F}(t){\mathop {=}\limits ^{def}}1-F(t)$, $I\{A\}$ denotes the indicator of a random event *A* and $1\le n\le N$.

Next, we verify the following identity (compare the first summand on the right side of (6)):

8 $$\begin{aligned} &\sum _{j=0}^{r}p_r^{j*}\frac{\lambda ^{j+1}}{j!}\int _{t=0}^{\infty }e^{-st}dt\int _{u=0}^{t} e^{-\lambda u}dG^{i*}(u)\int _{y=u}^{t}dy\int _{v=y-u}^{t-u}e^{-\lambda v}(u+v-y)^{j} H_{k+r}^X(t-u-v,m)dG(v)\nonumber \\ &=\sum _{j=0}^{r}p_r^{j*}\frac{\lambda ^{j+1}}{j!}\int _{u=0}^{\infty }e^{-(\lambda +s)u}\nonumber \\&dG^{i*}(u)\int _{y=u}^{\infty }dy\int _{v=y-u}^{\infty }(u+v-y)^{j}e^{-(\lambda +s)v}dG(v)\int _{t=u+v}^{\infty }e^{-s(t-u-v)}H_{k+r}^X(t-u-v,m)dt\nonumber \\ &=\sum _{j=0}^{r}p_r^{j*}\frac{\lambda ^{j+1}}{j!}\widehat{h}_{k+r}^X(s,m)\int _{u=0}^{\infty }e^{-(\lambda +s)u}dG^{i*}(u)\int _{v=0}^{\infty }e^{-(\lambda +s)v}dG(v)\int _{y=u}^{u+v}(u+v-y)^{j}dy\nonumber \\ &=\sum _{j=0}^{r}p_r^{j*}\frac{\lambda ^{j+1}}{(j+1)!}\widehat{h}_{k+r}^X(s,m)g^{i}(\lambda +s)\int _{0}^{\infty }e^{-(\lambda +s)v}v^{j+1}dG(v), \end{aligned}$$

where we apply the following definition:

9 $$\begin{aligned} &\widehat{h}_{j}^X(s,m){\mathop {=}\limits ^{def}}\int _{0}^{\infty }e^{-st}H_{j}^X(t,m)dt,\quad \textrm{Re}(s)>0, j \ge 0. \end{aligned}$$

Similarly, we derive

10 $$\begin{aligned} &\int _{t=0}^{\infty }e^{-st}dt\int _{u=0}^{t}\overline{G}(t-u)dG^{i*}(u)\int _{y=u}^{t}\lambda e^{-\lambda y}dy =\int _{u=0}^{\infty }e^{-(\lambda +s)u}dG^{i*}(u)\int _{t=u}^{\infty }\nonumber \\&\Bigl [e^{-s(t-u)}-e^{-(\lambda +s)(t-u)}\Bigr ]\overline{G}(t-u)dt\nonumber \\ &=g^{i}(\lambda +s)\Bigl [\frac{1-g(s)}{s}-\frac{1-g(\lambda +s)}{\lambda +s}\Bigr ]. \end{aligned}$$

Therefore, by defining

11 $$\begin{aligned} &a_{l}(s){\mathop {=}\limits ^{def}}\sum _{j=0}^{l}p_l^{j*}\int _{0}^{\infty }e^{-(\lambda +s)x}\frac{(\lambda x)^{j}}{j!}dF(x),\end{aligned}$$

12 $$\begin{aligned} &b_{l}(s){\mathop {=}\limits ^{def}}\frac{1}{1-g(\lambda +s)}\sum _{j=0}^{l}p_l^{j*}\int _{0}^{\infty }e^{-(\lambda +s)x}\frac{(\lambda x)^{j+1}}{(j+1)!}dG(x),\end{aligned}$$

13 $$\begin{aligned} &c(s){\mathop {=}\limits ^{def}}\sum _{k=N}^{\infty }p_k \cdot \frac{g(s)-g(\lambda +s)}{1-g(\lambda +s)} \end{aligned}$$

and

14 $$\begin{aligned} &d(s,m){\mathop {=}\limits ^{def}}\frac{\delta _{m,0}}{1-g(\lambda +s)}\Bigl [\frac{1-g(s)}{s}-\frac{1-g(\lambda +s)}{\lambda +s}\Bigr ], \end{aligned}$$

from (6)–(7) we obtain the following corresponding system for LTs:

15 $$\begin{aligned} &\widehat{h}_{0}^X(s,m)=\sum _{k=1}^{N-1}p_k\Bigl [\sum _{r=0}^{N-k-1}b_{r}(s)\widehat{h}_{k+r}^X(s,m)+\widehat{h}_{N}^X(s,m)\sum _{r=N-k}^{\infty }b_{r}(s)\Bigr ] +\widehat{h}_{N}^X(s,m)c(s)+d(s,m)+\frac{\delta _{m,0}}{\lambda +s} \end{aligned}$$

and

16 $$\begin{aligned} &\widehat{h}_{n}^X(s,m)=I\{m\ge 1\}\Bigl [\sum _{k=0}^{N-n-1}a_{k}(s)\widehat{h}_{n+k-1}^X(s,m-1)+\widehat{h}_{N-1}^X(s,m-1)\sum _{k=N-n}^{\infty }a_{k}(s)\Bigr ]+\delta _{m,0}\frac{1-f(s)}{s}, \end{aligned}$$

where $$1\le n\le N.$$

To avoid the inconvenience caused by $I\{\cdot \}$ indicator, we use now the introduced PGF (2) and the following definitions:

17 $$\begin{aligned} &\widetilde{a}_{j}(s,z){\mathop {=}\limits ^{def}}za_{j}(s),\end{aligned}$$

18 $$\begin{aligned} &\gamma (s,z){\mathop {=}\limits ^{def}}\sum _{m=0}^{\infty }z^{m} \Bigl (d(s,m)+\frac{\delta _{m,0}}{\lambda +s}\Bigr )=\frac{1-g(s)}{s[1-g(\lambda +s)]}. \end{aligned}$$

In consequence the Eq. (15)–(16) will transform into

19 $$\begin{aligned} &\widetilde{h}_{0}^X(s,z)=\sum _{k=1}^{N-1}p_k\Bigl [\sum _{r=0}^{N-k-1}b_{r}(s)\widetilde{h}_{k+r}^X(s,z)+\widetilde{h}_{N}^X(s,z)\sum _{r=N-k}^{\infty }b_{r}(s)\Bigr ] +\widetilde{h}_{N}^X(s,z)c(s)+\gamma (s,z),\end{aligned}$$

20 $$\begin{aligned} &\widetilde{h}_{n}^X(s,z)=\sum _{k=0}^{N-n-1}\widetilde{a}_{k}(s,z)\widetilde{h}_{n+k-1}^X(s,z)+\widetilde{h}_{N-1}^X(s,z)\sum _{k=N-n}^{\infty }\widetilde{a}_{k}(s,z)+\frac{1-f(s)}{s}, \end{aligned}$$

where $$1\le n\le N.$$

Finally by applying the following transformation into the system (19)–(20):

21 $$\begin{aligned} &\widetilde{u}_{n}^X(s,z){\mathop {=}\limits ^{def}}\widetilde{h}_{N-n}^X(s,z),\quad 0\le n\le N, \end{aligned}$$

for $0\le n\le N-1$, we obtain

22 $$\begin{aligned} &\widetilde{u}_{N}^X(s,z)=\sum _{k=1}^{N-1}p_k\Bigl [\sum _{r=0}^{N-k-1}b_{r}(s)\widetilde{u}_{N-k-r}^X(s,z)+\widetilde{u}_{0}^X(s,z)\sum _{r=N-k}^{\infty }b_{r}(s)\Bigr ] +\widetilde{u}_{0}^X(s,z)c(s)+\gamma (s,z), \end{aligned}$$

and

23 $$\begin{aligned} &\widetilde{u}_n^X(s,z)=\sum _{k=0}^{n-1}\widetilde{a}_k(s,z)\widetilde{u}_{n-k+1}^X(s,z)+\widetilde{u}_{1}^X(s,z)\sum _{k=n}^{\infty }\widetilde{a}_k(s,z)+\frac{1-f(s)}{s}. \end{aligned}$$

In the next steps, we transform Eq. (23)

24 $$\begin{aligned} &\widetilde{u}_n^X(s,z)=\sum _{k=0}^{n-1}\widetilde{a}_k(s,z)\widetilde{u}_{n-k+1}^X(s,z)+\widetilde{u}_{1}^X(s,z)\widetilde{a}_n(s,z)+\widetilde{u}_{1}^X(s,z)\sum _{k=n+1}^{\infty }\widetilde{a}_k(s,z)+\frac{1-f(s)}{s}\nonumber \\ &\widetilde{u}_n^X(s,z)=\sum _{k=0}^{n}\widetilde{a}_k(s,z)\widetilde{u}_{n-k+1}^X(s,z)+\widetilde{u}_{1}^X(s,z)\sum _{k=n+1}^{\infty }\widetilde{a}_k(s,z)+\frac{1-f(s)}{s}\nonumber \\ &\widetilde{u}_n^X(s,z)=\sum _{k=0}^{n+1}\widetilde{a}_k(s,z)\widetilde{u}_{n-k+1}^X(s,z)-\widetilde{u}_{0}^X(s,z)\widetilde{a}_{n+1}(s,z)+\widetilde{u}_{1}^X(s,z)\sum _{k=n+1}^{\infty }\widetilde{a}_k(s,z)+\frac{1-f(s)}{s}. \end{aligned}$$

Then, in the first sum on the right side of the Eq. (24), we substitute $\underline{k}=k-1$. To simplify the notation, we will omit the underscores in the notation of the new variable. We get

25 $$\begin{aligned} &\widetilde{u}_n^X(s,z)=\sum _{k=-1}^{n}\widetilde{a}_{k+1}(s,z)\widetilde{u}_{n-k}^X(s,z)-\widetilde{u}_{0}^X(s,z)\widetilde{a}_{n+1}(s,z)+\widetilde{u}_{1}^X(s,z)\sum _{k=n+1}^{\infty }\widetilde{a}_k(s,z)+\frac{1-f(s)}{s} \end{aligned}$$

and after moving the components to the appropriate sides of the equality sign

26 $$\begin{aligned} &\sum _{k=-1}^{n}\widetilde{a}_{k+1}(s,z)\widetilde{u}_{n-k}^X(s,z)-\widetilde{u}_{n}^X(s,z)=\widetilde{a}_{n+1}(s,z)\widetilde{u}_{0}^X(s,z)-\widetilde{u}_{1}^X(s,z)\sum _{k=n+1}^{\infty }\widetilde{a}_k(s,z)-\frac{1-f(s)}{s} \end{aligned}$$

we obtain:

27 $$\begin{aligned} &\sum _{k=-1}^{n}\widetilde{a}_{k+1}(s,z)\widetilde{u}_{n-k}^X(s,z)-\widetilde{u}_{n}^X(s,z)=\psi _{n}(s,z) \end{aligned}$$

where

28 $$\begin{aligned} &\psi _{n}(s,z){\mathop {=}\limits ^{def}}\widetilde{a}_{n+1}(s,z)\widetilde{u}_{0}^X(s,z)-\widetilde{u}_{1}^X(s,z)\sum _{k=n+1}^{\infty }\widetilde{a}_{k}(s,z)-\frac{1-f(s)}{s}. \end{aligned}$$

## Departure process solution in a compact form

Thanks to the specific form of (27), according to Theorem 1, we can write the following solution of the system (22)–(27):

29 $$\begin{aligned} &\widetilde{u}_{n}^X(s,z)=C(s,z)R_{n+1}(s,z)+\sum _{k=0}^{n}R_{n-k}(s,z)\psi _{k}(s,z),\quad n\ge 0, \end{aligned}$$

where $$R_{0}(s,z)=0, R_{1}(s,z)=\widetilde{a}_{0}^{-1}(s,z)$$ and

30 $$\begin{aligned} &R_{k+1}(s,z)=\frac{1}{\widetilde{a}_{0}(s,z)}\bigl (R_{k}(s,z)-\sum _{i=0}^{k}\widetilde{a}_{i+1}(s,z)R_{k-i}(s,z)\bigr ).\quad \end{aligned}$$

Further steps will lead us to find the representation for *C*(*s*, *z*). By substituting $n=0$ in (29) we obtain the equation

31 $$\begin{aligned} &C(s,z)=\widetilde{u}_{0}^X(s,z)\widetilde{a}_{0}(s,z). \end{aligned}$$

Next, by taking $n=0$ in (27), we obtain

32 $$\begin{aligned} &\widetilde{a}_{0}(s,z)\widetilde{u}_{1}^X(s,z)+\widetilde{a}_{1}(s,z)\widetilde{u}_{0}^X(s,z)-\widetilde{u}_{0}^X(s,z)=\psi _{0}(s,z). \end{aligned}$$

Taking into account that $\sum _{j=0}^{\infty }\widetilde{a}_{j}(s,z)=zf(s),$ from (28) we get

33 $$\begin{aligned} &\psi _{0}(s,z)=\widetilde{a}_{1}(s,z)\widetilde{u}_{0}^X(s,z)-\widetilde{u}_{1}^X(s,z)[zf(s)-\widetilde{a}_{0}(s,z)]-\frac{1-f(s)}{s}, \end{aligned}$$

thus, by applying (32), we eliminate $\widetilde{u}_{1}^X(s,z)$ in the form

34 $$\begin{aligned} &\widetilde{u}_{1}^X(s,z)=[zf(s)]^{-1}\Bigl [\widetilde{u}_{0}^X(s,z)-\frac{1-f(s)}{s}\Bigr ]. \end{aligned}$$

Having in mind the Eqs. (28), (31) and (34), from (29) we obtain

35 $$\begin{aligned} &\widetilde{u}_{n}^X(s,z)=\widetilde{a}_{0}(s,z)R_{n+1}(s,z)\widetilde{u}_{0}^X(s,z) +\sum _{k=0}^{n}R_{n-k}(s,z)\Bigl [\widetilde{a}_{k+1}(s,z)\widetilde{u}_{0}^X(s,z) -\widetilde{u}_{1}^X(s,z)\sum _{i=k+1}^{\infty }\widetilde{a}_{i}(s,z)-\frac{1-f(s)}{s}\Bigr ]=\nonumber \\ &\widetilde{a}_{0}(s,z)R_{n+1}(s,z)\widetilde{u}_{0}^X(s,z)+\sum _{k=0}^{n}R_{n-k}(s,z)\nonumber \\&\Bigl [\widetilde{a}_{k+1}(s,z)\widetilde{u}_{0}^X(s,z) -\bigl (zf(s)\bigr )^{-1}\Bigl (\widetilde{u}_{0}^X(s,z)-\frac{1-f(s)}{s}\Bigr )\sum _{i=k+1}^{\infty }\widetilde{a}_{i}(s,z)-\frac{1-f(s)}{s}\Bigr ]. \end{aligned}$$

The above representation can be written using simplified notation, namely

36 $$\begin{aligned} &\widetilde{u}_{n}^X(s,z)=A_{n}(s,z)\widetilde{u}_{0}^X(s,z)+B_{n}(s,z), \end{aligned}$$

where

37 $$\begin{aligned} &A_{n}(s,z){\mathop {=}\limits ^{def}}R_{n+1}(s,z)\widetilde{a}_{0}(s,z) +\sum _{k=0}^{n}R_{n-k}(s,z)\Bigl [\widetilde{a}_{k+1}(s,z)-\bigl (zf(s)\bigr )^{-1}\sum _{i=k+1}^{\infty }\widetilde{a}_{i}(s,z)\Bigr ] \end{aligned}$$

and

38 $$\begin{aligned} &B_{n}(s,z){\mathop {=}\limits ^{def}}\frac{1-f(s)}{zsf(s)}\sum _{k=0}^{n}R_{n-k}(s,z)\Bigl (\sum _{i=k+1}^{\infty }\widetilde{a}_{i}(s,z)-zf(s)\Bigr ). \end{aligned}$$

Finally by substituting the representation (36) into (22), we obtain

39 $$\begin{aligned} &\widetilde{u}_{N}^X(s,z)=A_{N}(s,z)\widetilde{u}_{0}^X(s,z)+B_{N}(s,z)=\sum _{k=1}^{N-1}p_k\Bigl [\sum _{r=0}^{N-k-1}b_{r}(s)\bigl (A_{N-k-r}(s,z)\widetilde{u}_{0}^X(s,z)+B_{N-k-r}(s,z)\bigr )\nonumber \\ &+\widetilde{u}_{0}^X(s,z)\sum _{r=N-k}^{\infty }b_{r}(s)\Bigr ]+\widetilde{u}_{0}^X(s,z)c(s)+\gamma (s,z) \end{aligned}$$

and hence we eliminate $\widetilde{u}_{0}^X(s,z)$ as follows:

40 $$\begin{aligned} &\widetilde{u}_{0}^X(s,z)= \frac{\sum _{k=1}^{N-1}p_k\sum _{r=0}^{N-k-1}b_{r}(s)B_{N-k-r}(s,z)-B_{N}(s,z)+\gamma (s,z)}{A_{N}(s,z)-c(s)-\sum _{k=1}^{N-1}p_k\Bigl [\sum _{r=0}^{N-k-1}b_{r}(s)A_{N-k-r}(s,z)+\sum _{r=N-k}^{\infty }b_{r}(s)\Bigr ]}. \end{aligned}$$

Taking into consideration the formulae (21), (36) and (40), we state the following:

### Theorem 2

In the $M^X/G/1/N$-type queueing model with batch arrivals and MVP, the PGF of LT (mixed double transform) of the conditional probability distribution of the number of departures up to the fixed time *t* (i.e., departure process at time *t*) $\widetilde{h}_{n}^X(s,z)$ can be found using the following formulae:

41 $$\begin{aligned} &\widetilde{h}_{n}^X(s,z)=B_{N-n}(s,z)+A_{N-n}(s,z)\cdot \frac{\sum _{k=1}^{N-1}p_k\sum _{r=0}^{N-k-1}b_{r}(s)B_{N-k-r}(s,z)-B_{N}(s,z)+\gamma (s,z)}{A_{N}(s,z)-c(s)-\sum _{k=1}^{N-1}p_k\Bigl [\sum _{r=0}^{N-k-1}b_{r}(s)A_{N-k-r}(s,z)+\sum _{r=N-k}^{\infty }b_{r}(s)\Bigr ]}, \end{aligned}$$

where the formulae for $b_{k}(s),$ *c*(*s*),  $\gamma (s,z),$ $A_{k}(s,z)$ and $B_{k}(s,z)$ are given in (12), (13), (18), (37) and (38), respectively.

As Theorem 2 shows, the distribution of the number of packets served up to a fixed moment in the considered queueing model is significantly dependent on the initial state of the buffer (i.e., on the value of n in the condition $Y(0)=n$). It is, of course, possible to abandon this condition, but then it would be necessary to determine in what state the system starts working. One of two assumptions could be made here. Either the system starts working with an empty buffer ($n=0$) or with a specified probability the buffer state is equal to a specified value. The adoption of the second assumption would involve the necessity of defining an additional sequence $q_k$ for $k=0,..., N$, where $q_k$ means that at the initial moment, the system contains exactly *k* packets.

## The mean number of departures

A numerical algorithm capable of directly calculating $H_n^X(t,m)$ is presented in^37^. The method involves the use of mixed transforms and provides a detailed framework for reconstructing $H_n^X(t,m)$ from its transform representation. However, this approach is highly computationally intensive, as it requires complex numerical computations, including the evaluation of multiple infinite series and sums using techniques such as Euler’s summation. Due to the significant computational burden of this direct method, our research focuses on an alternative approach to calculate the mean number of departures, which avoids the complexity of direct numerical computation while still providing valuable insights into the system’s behavior.

As a consequence of Theorem 2, we can find the representation for the average number of successful departures up to fixed time epoch *t*,  conditioned by the number of packets initially accumulated in the buffer.

Let us introduce the following notation:

42 $$\begin{aligned} &{\textbf{E}}H_n^X(t){\mathop {=}\limits ^{def}}{\textbf{E}^X}\{h(t)\,|\,Y(0)=n\}=\sum _{m=0}^{\infty }m\cdot {\textbf{P}}\{h(t)=m\,|\,Y(0)=n\}. \end{aligned}$$

Observe that

43 $$\begin{aligned} &\textrm{LT}^{-1}\Bigl [\frac{\partial }{\partial z}\widetilde{h}_{n}^X(s,z)\bigr |_{z=1}\Bigr ]=\textrm{LT}^{-1}\bigg [\frac{\partial }{\partial z}\Big [\int _{t=0}^{\infty }e^{-st}\sum _{m=0}^{\infty }z^m\cdot {\textbf{P}}\{h(t)=m\,|\,Y(0)=n\}dt\Big ]\bigg |_{z=1}\bigg ]=\nonumber \\ &\textrm{LT}^{-1}\bigg [\Big [\int _{t=0}^{\infty }e^{-st}\sum _{m=0}^{\infty }mz^{m-1}\cdot {\textbf{P}}\{h(t)=m\,|\,Y(0)=n\}dt\Big ]\bigg |_{z=1}\bigg ]= \textrm{LT}^{-1}\Big [\int _{t=0}^{\infty }e^{-st}\sum _{m=0}^{\infty }m\cdot {\textbf{P}}\{h(t)=m\,|\,Y(0)=n\}dt\Big ]\nonumber \\ &={\textbf{E}}H_n^X(t), \end{aligned}$$

and therefore

44 $$\begin{aligned} &{\textbf{E}^X}\{h(t)\,|\,Y(0)=n\}=\textrm{LT}^{-1}\Bigl [\frac{\partial }{\partial z}\widetilde{h}_{n}^X(s,z)\bigr |_{z=1}\Bigr ], \end{aligned}$$

where the notation $\textrm{LT}^{-1}$ denotes the inverse LT operator.

## Numerical examples

In this section, we illustrate the theoretical results using numerical examples. First, we perform a comparative analysis of numerical calculations made in *Mathematica 12.1* and simulations made in *Objective Modular Network Testbed in C++* (OMNeT++). The OMNeT++ environment was designed for computer network simulation (see, e.g.,^38^). Secondly, we discuss the dependence of the number of departures counted to fixed time epoch *t* on server vacation duration, the initial number of packets, the intensity of arrivals, and the processing speed.

Assume that packets of average sizes of 100 B arrive at the wireless sensor network node individually or in batches according to the Poisson process with rate $\lambda$. They are being individually processed with exponentially distributed service time with rate $\mu ,$ accordingly to FIFO service discipline. Finally, we also assume that a multiple vacation period consists of exponentially distributed independent server vacations, each with a mean $1/\lambda _v$. To obtain explicit representations for the Laplace mixed double transform of the conditional probability distribution of the number of departures up to fixed time *t*, we use the formula from Theorem 2. Next, we use the formula (44) to calculate the mean number of successful departures. The $\textrm{LT}^{-1}$ operator is calculated using the procedures of numerical Laplace transform inversion. We compare the results of two algorithms used in the literature: the Abate-Choudhury-Whitt algorithm presented in^39^, and the Gaver-Stehfest algorithm proposed in^40,41^, which is a combination of two approaches given in^42^ and^43^. By choosing the better one, we present the obtained results in successive subsections and figures. In the rest of the chapter, all presented results were generated for a model whose capacity, defined by the value *N*, is ten packets (the accumulation buffer can store up to nine packets while one packet is being served).

### Average queue utilization

In queuing theory, there is a duality in the way of calculating the system load due to a single or group inflow of requests. As written in the already cited monograph^35^, the system load $\varrho$ for queuing a simple Poisson process is defined as the so-called offered load, i.e. the product of the input stream intensity value $\lambda$ and the average service time $\mu$, namely $\varrho =\frac{\lambda }{\mu }$. The way of calculating the load changes in the case of queuing a compound Poisson process (e.g.^35^, formula (2.72), page 49) must take into account the average size of the group $\varepsilon$. The presented duality is a standard approach to defining the load. In the rest of the article, we will use the term average fullness metric $\kappa _b$ to call the product of the average packet size $\varepsilon$ [B] and the system load $\varrho$ (i.e. $\kappa _b=\varepsilon \cdot \varrho$), while maintaining a definition of system load as the ratio of the number of incomes of packets (regardless the size of a batch) to the number of requests handled per unit of time. The term average fullness metric is inspired by:

- The manuscript^44^, where the average fullness of the virtual buffer is used in the DVB-H decoder simulation.;
- The thesis^45^, that introduces the average fullness of the buffer in the conceptual model of DASH adaptive algorithms;
- The article^46^ proposing a system for handling video streaming over a wireless network, which is based on adaptive management of the playback buffer on the client’s HTTP protocol side. It uses the concept of regression to predict future buffer fill for a fixed time interval. The feedback sent to the server depends on the value of the average buffer fullness and its trend over a given time interval.

In the following experiments, we proposed a common scenario, assuming that despite the differences in values of the input stream intensity and the average service times, due to the average group size $\varepsilon =1.75,$ the wireless sensor network node will theoretically be overloaded for most of the time, as the values of $\kappa _b \in \{1.3125, 1.4, 1.75, 2.1875\}$. This scenario corresponds to a hypothetical situation with challenging conditions of a potential disaster occurrence. Sensor network nodes, similar to all other computer network devices, are designed to sustain undisturbed transmission in an environment with a balanced packet traffic load. If several nodes support the traffic in the network, the network is designed to distribute network traffic as proportionally as possible between the nodes involved in packet transmission. If, due to an unforeseen situation, one of the nodes fails or is destroyed, then the number of remaining operating nodes must take over the network traffic, which may lead to their overload for a certain period. After a node failure occurs, the wireless sensor network is reconfigured, and each of the remaining nodes starts handling packets in excess network traffic. The simulation and numerical examples presented in the manuscript are intended to reflect this situation from the perspective of a single sensor network node. This constitutes a kind of stress test of the operation of a wireless sensor network node.

### Comparative analysis of numerical calculations and simulations

Simulation tests were carried out on samples of ten thousand repetitions of starting the packet queueing process. For each sample, a unique seed was drawn and used to initialize the generator of pseudo-random numbers responsible for the randomness of the moments of arrival and departures of packets, as well as the length of individual vacation periods. In the simulations, the registration of the number of handled packets was enabled, performed with a time step of $t_c=0.0005$ seconds and moments $k\cdot t_c=t\in [0,0.2]$ for $k=0;1;2;...;400,$ in which the current number of handled packets was recorded.

**Fig. 1:**
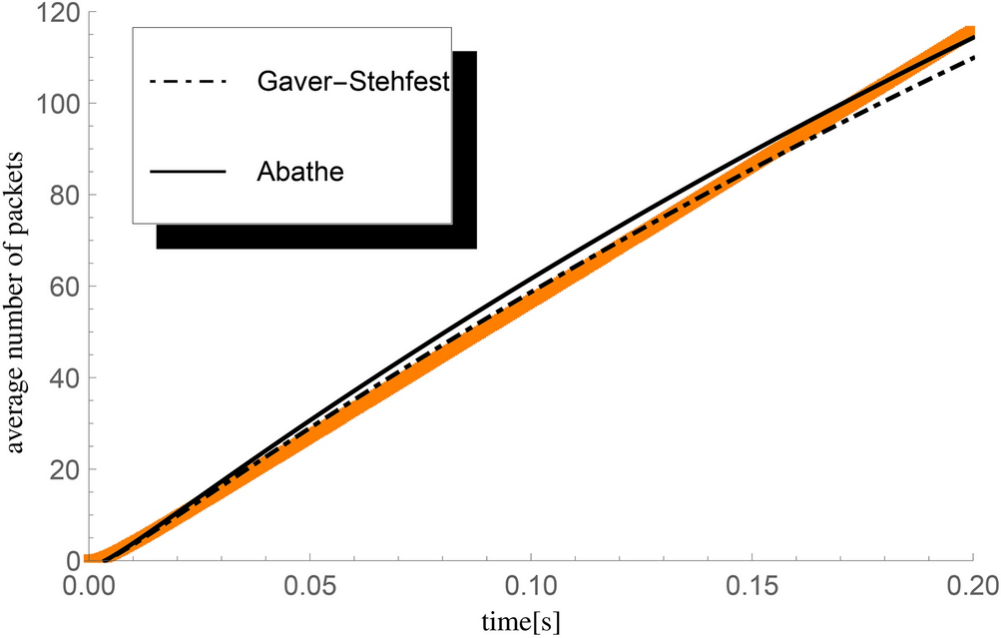
Comparison of the average number of packets ${\textbf{E}}H_0^X(t)$ handled until *t* for the compound Poisson process, obtained: 1) by numerical calculations – black lines on the graph, the dashed one obtained for the Gaver-Stehfest algorithm, and the solid one for the Abate-Choudhury-Whitt algorithm of inverting the Laplace transform (abbreviated as ’Abathe’); 2) as a statistical result of simulation tests - orange squares on the chart.

The remaining parameters of the considered queueing model are as follows: initial number of packets present in the system $n=0$, intensity of the input flow of packets $\lambda =600$ packets/s, service speed $\mu =600$ packets/s, CDF $G(\cdot )$ parameter $\lambda _v=1000$ (then a single vacation period lasts on average $t_p=0.001$ s). The last parameter in the model, related to the group arrival of packets in a compound Poisson process, is the sequence of probabilities $(p_k)=\{0.5,0.25,0.25,0,...\},$ whose form gives the average group size $\varepsilon =1.75,$ whereas system load $\varrho =1$ so the average fullness metric $\kappa _b=1.75$. Let us briefly describe the relationship between the size of basic volume unit 100 B and the intensity of packet arrival $\lambda$. Firstly, it’s important to clarify that $\lambda$ represents the intensity of the arrival process, indicating the average rate at which packets enter the system per unit time. However, in this system, arrivals occur in batches, and each batch contains a certain number of packets, each with a volume that is a multiple of the basic volume unit (100 B, 200 B, 300 B, etc.). For example, we equate a single packet of size 300 B incoming according to the intensity $\lambda$ with a batch of three packets of 100 B each. From the provided sequence of probabilities $(p_k)$, the probability of such a packet entering the system is $p_3=0.25$.

A comparative analysis of numerical calculations and simulations is presented in Fig. 1.

The maximum differences in the values of the average number of handled packets, determined at times $k\cdot t_c$, between numerical calculations and simulation results, are:

- 5.9372 for the Gaver-Stehfest algorithm for inverting the Laplace transform,
- 4.92585 for the Abate-Choudhury-Whitt algorithm for inverting the Laplace transform.

The following examples present the results obtained using a more accurate Abate-Choudhury-Whitt algorithm of inverting the Laplace transform.

### Impact of vacation duration

**Fig. 2:**
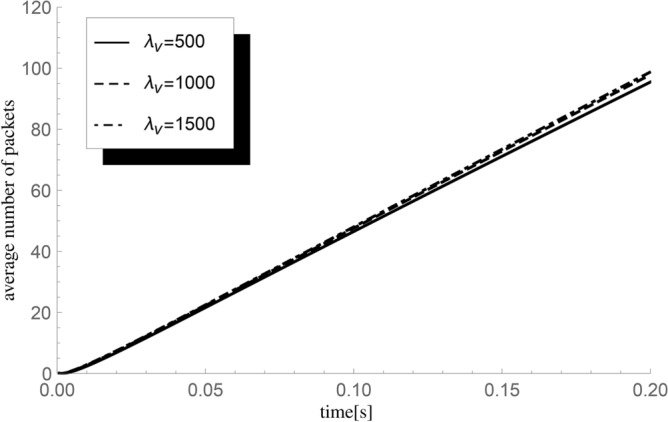
The impact of changes in the value of the inverse of the mean vacation duration $\lambda _{v}$ on the average value ${\textbf{E}}H_0^X(t)$ of the number of packets handled until *t* for $\lambda =375$ packets/s and $\mu =500$ packets/s (system load $\varrho =0.75$, average fullness metric $\kappa _b=1.3125$).

In the first example, we visualize the dependence of the mean number of successful departures up to fixed time *t* on the single server vacation duration. Single vacation period parameter takes on values $\lambda _v=500, 1000$ and 1500 (which corresponds to the average lengths of a single vacation period $t_p$ being 0.002, 0.001, and 0.0006(6) seconds, respectively). The remaining system parameters are $\lambda =375$ packets/s, $n=0$, $\mu =500$ packets/s, average group size $\varepsilon =1.75,$ and sequence of probabilities $(p_k)=\{0.5,0.25,0.25,0,...\}.$ The results are presented in Fig. 2.

### Impact of the initial number of packets

**Fig. 3:**
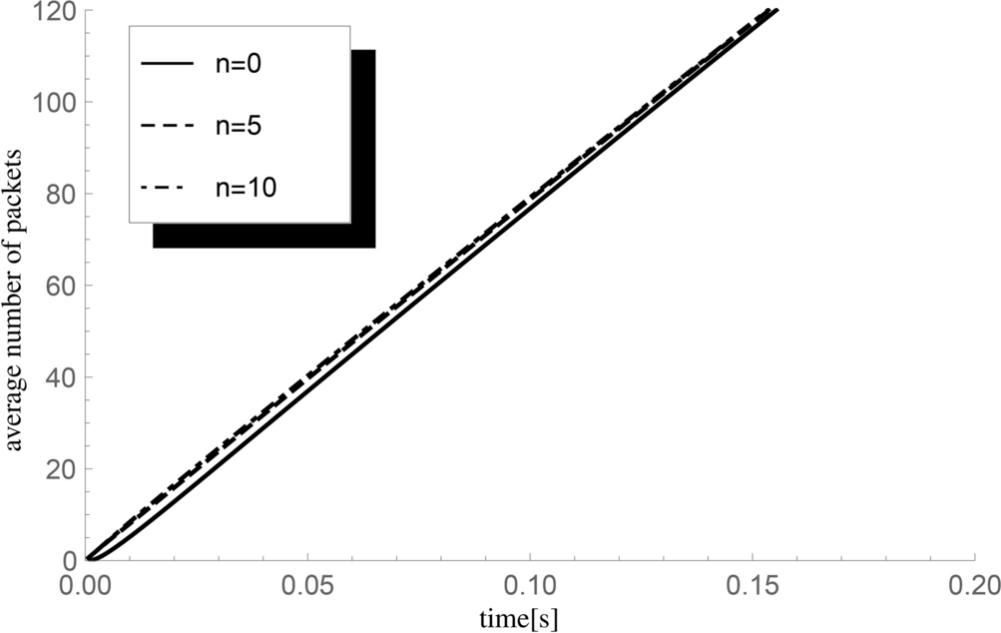
The impact of changes in the value of the number of packets *n* present inside the system at time $t=0$ on the average value ${\textbf{E}}H_n^X(t)$ of the number of packets served until *t* for $\lambda =600$ packets/s and $\mu =800$ packets/s (system load $\varrho =0.75$, average fullness metric $\kappa _b=1.3125$).

Considering the following experiment, we will analyse the impact of changes in the initial value *n* of the number of packets on the average value of the number of packets handled until *t*. We will use the following inflow and packet service parameter combinations: $(\lambda ,\mu )=(600,800)$. The rest of the parameters remain unchanged: the inverse of the scale parameter $\lambda _v=1000$ (then a single vacation period lasts on average $t_p=0.001$ s), average group size $\varepsilon =1.75,$ sequence of probabilities $(p_k)=\{0.5,0.25,0.25,0,...\}.$ The results are presented in Fig. 3.

### Impact of arrival intensity

**Fig. 4:**
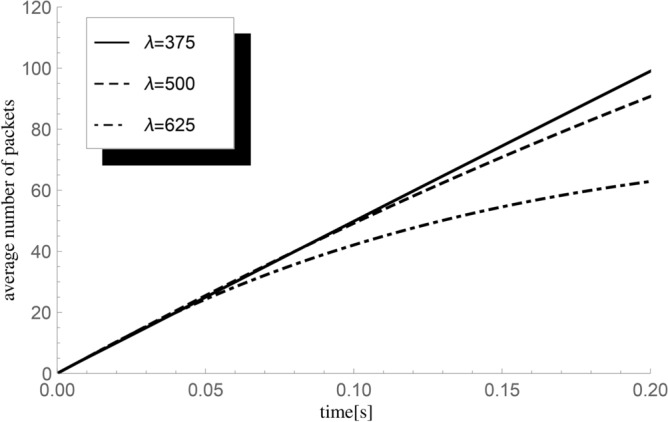
The impact of changes in the intensity of the inflow of requests $\lambda$ on the average value ${\textbf{E}}H_5^X(t)$ of the number of packets handled until time *t* for $\mu =500$ packets/s.

Next, we visualize the dependence of the mean number of successful departures up to fixed time *t* on the intensity of packet arrival rate $\lambda =375,500$ and 625 packets/s. The initial buffer state $n=5,$ the processing rate $\mu =500$ packets/s, and the exponentially distributed server vacation parameter $\lambda _v=1000.$ The results are presented in Fig. 4.

### Impact of processing rate

**Fig. 5:**
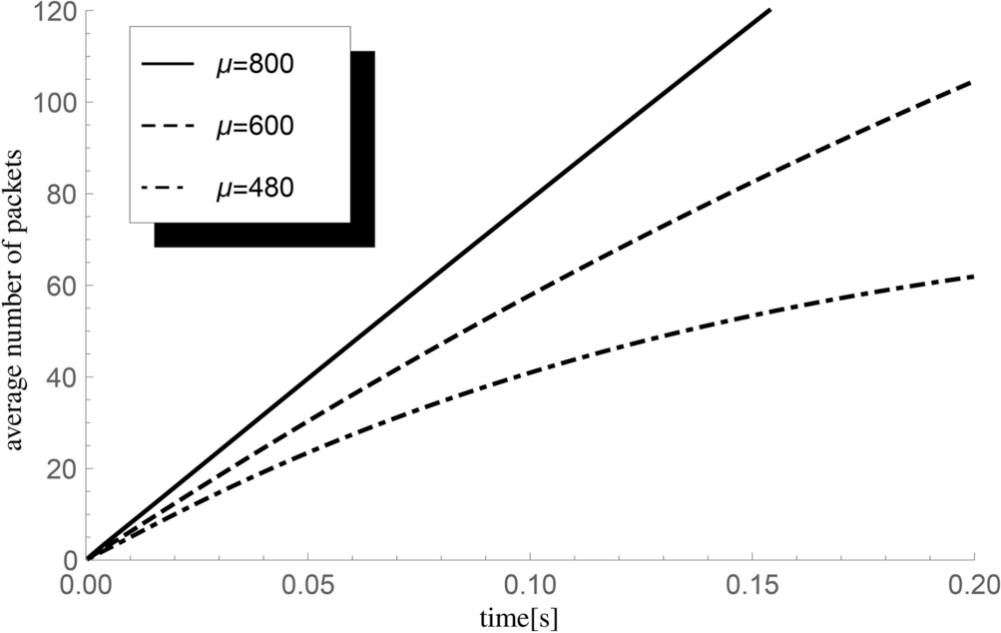
The impact of changes in the intensity of $\mu$ on the average value ${\textbf{E}}H_5^X(t)$ of the number of packets handled until time *t* for $\lambda =600$ packets/s.

Finally, we investigate the impact on the mean number of successful departures up to fixed time *t* of the service speed expressed by three exponential processing rates $\mu =800, 600$ and 480 packets/s, where the arrival rate $\lambda =600$ packets/s, the initial buffer state $n=5,$ and $\lambda _v=1000.$ The results are visualized in Fig. 5.

## Conclusions

Some elements of the analytical approach proposed in this paper can be applied to other stochastic characteristics of the M/G/1 type queueing model with a multiple vacation mechanism in the transient state. However, there are also very important differences. In^47^, the probability distribution of the non-stationary queueing delay is investigated; however, only in the case in which successive single vacations started by the service station have a fixed constant duration. Transient queue-size distribution is studied in^48^, where the representation for the single Laplace transform is obtained and, hence, the appropriate stationary result by applying the Tauberian theorem. Lastly, one can find in^49^ the analysis of the time to the first buffer overflow, where also the representation for the one-dimensional Laplace transform is derived.

The analysis of the obtained average values of the number of packet handled until a fixed time *t* (in transient state) carried out in the article gives an insight into the characteristics of the counting process of handled packets in the queueing model with the multiple vacation policy. The presented graphs show the essential impact of changes in the values of parameters describing the intensity of the inflow of requests and the service speed, the initial number of packets accumulated in the buffer, and the length of the vacation period.

Concluding, as one can observe, the most significant impact on the behavior of the mean number of packets processed up to the fixed time *t* has the parameter describing the service speed. The arrival rate $\lambda$ also significantly impacts the obtained curves. The slightest impact is observed in the case of changes in the value of the parameter $\lambda _v$ describing the distribution of the single vacation duration and changes in the initial value of the number of packets *n* present in the system.

Overall, the research findings prove it possible to predict how many packets will be processed by a single wireless sensor network node, even when it is overloaded. This allows for a more in-depth understanding of the impact of extreme conditions (significant failures or disasters) on the node’s operation in an environment with reduced processing capacity. Our research’s insights provide the basis for developing strategies to enhance system performance and resilience in the face of potential failures or disasters in wide-area sensor networks. The possibility to perform predictive analysis of departure dynamics in a wireless sensor network node operating under multiple vacation policy, and under excess network traffic conditions aids in predicting the consequences of failure of some part of the sensor network. In turn, it allows for the development of protection against such situations, e.g., by refining the mechanisms of a sensor network recovery from a failure, including identifying the network’s bottlenecks and determining the number of nodes necessary to maintain transmission in the network at the assumed level.

## Data Availability

All data generated or analyzed during this study are included in this published article.
